# Oxidative Stress Induced by Air Pollution

**DOI:** 10.3390/antiox13111393

**Published:** 2024-11-15

**Authors:** Yasuhiro Yoshida

**Affiliations:** Department of Immunology and Parasitology, School of Medicine, University of Occupational and Environmental Health, 1-1 Iseigaoka, Yahatanishi-ku, Kitakyushu 807-8555, Japan; freude@med.uoeh-u.ac.jp

## 1. Introduction

In 2021, the World Health Organization issued new guidelines on particulate matter (PM), including PM2.5, and highlighted associated risks (WHO global air quality guidelines). Among the various types of particles, fine-particle substances (PM2.5), as well as industrial/pharmaceutical nanoparticles, differ in terms of properties and pose serious health risks. Epidemiological studies indicate that exposure to particulate matter is associated with various adverse health effects [1]. PM2.5 exposure is linked to cardiovascular diseases, respiratory disorders, and lung cancer, especially in large cities. This exposure induces inflammation and produces reactive oxygen species (ROS), leading to oxidative stress, which affects various cells and can cause cell death. Both acute and chronic inflammation contribute to these biological effects.

Many studies have explored the relationship between environmental particulates and health effects through cell and animal experiments, but many questions remain unanswered. This Special Issue will delve into the oxidative stress caused by these pollutants and cover topics related to urgent issues in this field.

## 2. Overview of Published Articles

PM2.5 sources include anthropogenic sources and natural sources such as volcanoes. Anthropogenic sources include facilities that generate soot, such as incinerators; facilities that generate dust; and automobiles. Exposure to PM2.5, especially in urban areas, is of great concern in terms of health effects. Sanchez-Rodriguez, L. et al. studied the relationship between traffic-related air pollution (TRAP) and oxidative stress (Contribution 1). This study revealed the effect of traffic density on plasma metabolites and urinary oxidative stress biomarkers, indicating that oxidative stress could possibly play a role as an intermediate factor in metabolic changes. PM2.5 exposure is not limited to the living environment, and exposure in the workplace is a topic that has also been attracting attention. Bellisario, V. et al. studied occupational exposure to metal nanomaterials and suggested a possible relationship between chemical composition and oxidative stress biomarkers (Contribution 2). They showed that occupational exposure to nanomaterials (NM) in particular was associated with urinary silica (Si) and titanium (Ti) concentrations and increased oxidative stress.

The skin and eyes may act as the first defense mechanism against PM2.5. Two reports on substances that could have a protective effect against skin damage are included in this Special Issue. Liyanage, N.M. et al. demonstrated that the cryonasterol-rich hexane fraction (CRHF2) from *Caulerpa racemosa* has a protective effect at the cell level and in zebrafish (Contribution 3). CRHF2 reduced intracellular and mitochondrial ROS levels, inhibited PM-induced apoptosis, downregulated the expression of apoptosis signaling pathway proteins, and reduced the accumulation of sub-G1 cells in a dose-dependent manner. Zhen, A.X. et al. also showed that the marine algae-derived compound 3-bromo-4,5-dihydroxybenzaldehyde (3-BDB) suppresses skin damage (Contribution 4). They demonstrated that 3-BDB suppresses ROS generation, mitochondrial dysfunction, DNA damage, and cellular senescence in vitro and in vivo. Both of these compounds possess the ability to suppress ROS produced by mitochondria, a discovery that raises hopes for drug development. On the other hand, there is a strong demand for in vitro research methods to develop such drugs and inhibitors, which is a global trend in line with the 3R principle (refinement, reduction, and replacement) [2]. Zeng, Z. et al. demonstrated the construction of an in vitro system to investigate the mechanism of inflammation caused by particles in the eyes. This study demonstrates that PM induces inflammation in a co-culture system of corneal epithelial cells and neutrophils and that ROS and NF-κB are involved in the mechanism (Contribution 5). The authors of this study therefore propose that NF-κB is a candidate molecular target for ROS-dependent inflammation inhibitors. From this antioxidant perspective, the search for therapeutic drugs that suppress the inflammation, damage, and diseases caused by PM may provide a guideline for the future.

Once particles enter the body, their impact on the respiratory system, especially the lungs, is of concern. Hammond, J. et al. investigated the cytotoxicity of PM2.5 collected from three different cities (Lancaster and Birmingham in the UK and Mexico City, Mexico) using lung epithelial cells (Calu-3) (Contribution 6). Samples from all cities induced the generation of ROS and the production of inflammatory cytokines. It is interesting to note that the authors pointed out that mass-based PM (particulate matter) restrictions do not fully reflect the composition and health effects of PM specific to each region. Therefore, it is important to develop and introduce new biologically relevant indicators and regionally appropriate regulations. In addition, Kim, T.Y. et al. demonstrated in experiments using mice that the aqueous extract of *Codium fragile* could potentially be a functional food or pharmaceutical ingredient (Contribution 7). *Codium fragile* suppresses pulmonary mitochondrial dysfunction by regulating ROS content and mitochondrial membrane potential levels. They pointed out the involvement of the TLR/TGF-β pathway as a mechanism. It is interesting to note that this is consistent with the involvement of NF-κB pointed out by Zeng et al. (Contribution 5).

Another important question is “Does PM affect systems other than the respiratory and cardiovascular systems?” From this perspective, the study by Park, J. et al., focusing on the effects of PM on skeletal muscle and exercise, is intriguing (Contribution 8). In a mouse study, these authors demonstrated that PM exacerbated oxidative stress and inflammation in skeletal muscle and mitochondria both at rest and during exercise, and a significant increase in the level of in vivo mitophagy was observed in the PM group.

The effects of PM on our working generation are a matter of great concern, as are the effects that the next generation could face. In a retrospective cohort study, Park, S. et al. demonstrate that indoor PM2.5 concentrations in their low birth weight (LBW) group were significantly higher than those in the normal birth weight (NBW) group (Contribution 9). In a prospective study, 8-hydroxy-2-deoxyguanosine was significantly higher in the high PM2.5 group, and these results suggest a relationship between PM2.5, oxidative stress, and fetal development.

The effects of these particles may be linked to our everyday lifestyle. Ossoli, A. et al. present a study evaluating the effect of short-term exposure to PM on high-density lipoprotein (HDL) function and the modifying effect of body mass index (BMI) (Contribution 10). A positive association between PM10 exposure and NO production was observed in participants with normal BMI but not in participants with high BMI. This suggests that an increase in BMI may cause the loss of HDL function’s compensatory response to PM exposure and that PM-induced endothelial dysfunction may have a greater impact on obese individuals, raising a warning about the increased environmental risks to obese individuals.

## 3. Conclusions

This Special Issue highlights the diverse health risks associated with PM exposure, showing that PM2.5 and related pollutants contribute to oxidative stress and inflammation across multiple biological systems. The studies included within reveal PM’s impact on metabolic and occupational health, respiratory function, and fetal development, with notable effects in urban and workplace settings. Promising protective strategies, including the use of marine algae-derived compounds, have shown potential in reducing PM-induced cellular damage.

In this Special Issue, the need for targeted regulatory measures and biologically relevant health indicators is emphasized, as is the importance of mitigating environmental risks for vulnerable groups, including those with higher BMI. Together, these findings underscore the importance of reducing PM exposure to protect public health.
